# A survey on worries of pregnant women - testing the German version of the Cambridge Worry Scale

**DOI:** 10.1186/1471-2458-9-490

**Published:** 2009-12-28

**Authors:** Juliana J Petersen, Michael A Paulitsch, Corina Guethlin, Jochen Gensichen, Albrecht Jahn

**Affiliations:** 1 Institute for General Practice, Johann-Wolfgang Goethe-University Frankfurt/Main, Theodor-Stern-Kai 7, 60590 Frankfurt/Main, Germany; 2 Institute for General Practice, University Hospital Jena, Friedrich-Schiller-University, Jena, Germany; 3 Department of Tropical Hygiene and Public Health, Ruprecht-Karls-University, Im Neuenheimer Feld 324, 69120 Heidelberg, Germany

## Abstract

**Background:**

Pregnancy is a transition period in a woman's life characterized by increased worries and anxiety. The Cambridge Worry Scale (CWS) was developed to assess the content and extent of maternal worries in pregnancy. It has been increasingly used in studies over recent years. However, a German version has not yet been developed and validated.

The aim of this study was (1) to assess the extent and content of worries in pregnancy on a sample of women in Germany using a translated and adapted version of the Cambridge Worry Scale, and (2) to evaluate the psychometric properties of the German version.

**Methods:**

We conducted a cross-sectional study and enrolled 344 pregnant women in the federal state of Baden-Württemberg, Germany. Women filled out structured questionnaires that contained the CWS, the Spielberger-State-Trait-Anxiety Inventory (STAI), as well as questions on their obstetric history. Antenatal records were also analyzed.

**Results:**

The CWS was well understood and easy to fill in. The major worries referred to the process of giving birth (CWS mean value 2.26) and the possibility that something might be wrong with the baby (1.99), followed by coping with the new baby (1.57), going to hospital (1.29) and the possibility of going into labour too early (1.28). The internal consistency of the scale (0.80) was satisfactory, and we found a four-factor structure, similar to previous studies. Tests of convergent validity showed that the German CWS represents a different construct compared with state and trait anxiety but has the desired overlap.

**Conclusions:**

The German CWS has satisfactory psychometric properties. It represents a valuable tool for use in scientific studies and is likely to be useful also to clinicians.

## Background

Pregnancy is a transition period in a woman's life characterized by physiologic and psychological changes. In this period, many women report increased worries and anxiety [1]. Enhanced levels of anxiety during pregnancy may affect maternal blood flow [2] and contribute to adverse obstetric, fetal and neonatal outcomes [3,4]. Risk factors for increased worries and anxiety are, for instance, single status and nulliparity [1]. Additionally, positive results from genetic screening or a prenatal diagnosis of fetal malformation increase anxiety in pregnant women [5,6]. Even the suspicion of fetal abnormality conduces to strong feelings of anxiety, or worries [7,8]. Thus, the assessment of anxiety and worries in pregnancy is an important issue that warrants adequate and validated instruments for assessment.

A widely used instrument for measuring anxiety is the Spielberger-State-Trait-Anxiety Inventory (STAI) [9], which has been used in numerous studies on pregnant women. Grant et al., for instance, examined the course of maternal anxiety across the transition to parenthood by using the STAI, whereas Fertl et al. used the STAI on a sample of women who had experienced prior miscarriages [10,11]. The STAI is a validated instrument that is fairly short and easy to fill in. However, it should be taken into account that the STAI measures the extent of anxiety at a particular point in time and provides no information on what the pregnant woman is anxious about [12]. Furthermore, a study by Hundley et al. indicated that the STAI may be unstable around the time of delivery [13]. Green et al. developed the Cambridge Worry Scale (CWS) with the aim of assessing both the content and the extent of women's worries during pregnancy. The focus was on pregnancy-related and more general worries, which were regarded as non-pathological [12]. The CWS was developed for use in the "Cambridge Prenatal Screening Study" on a sample of 1072 women that was broadly representative of the UK childbearing population with regard to age, parity, education and socio-economic class [14]. The original English version of the CWS demonstrated satisfactory reliability and validity [12], which was also confirmed for its use in early pregnancy [15]. A Swedish version of the CWS has been validated by Georgsson Öhman et al. [16]. The importance of an instrument measuring worries in pregnancy is evidenced by the increasing use of the CWS in research projects over recent years [17-24].

Furthermore, the CWS has been adapted for use with other populations, such as parents of disabled children [25] and women with a family history of cancer [26].

To our knowledge, a German version of the CWS is not yet available. The aim of this study was to assess the extent and content of worries in pregnancy on a sample of women in Germany using a translated and adapted version of the Cambridge Worry Scale, and to evaluate the psychometric properties of the German version.

## Methods

### Study setting and participants

We conducted a cross-sectional study, comprising pregnant women who attended antenatal classes in the towns of Mannheim and Heidelberg, and the Rhein-Neckar region of Baden-Württemberg, Germany. Antenatal classes represent an add-on to antenatal care and are usually provided by midwives. The cost of the classes are covered by medical insurance and most pregnant women take part in them [27]. Information on the midwives offering antenatal classes were taken from an official list provided by the German federation of midwives (German federation of midwives, branch of Baden-Württemberg. *List of midwives in the Rhein-Neckar area*) and by directly contacting hospitals or private practices not registered in the list. Of 38 eligible midwives, 34 (89.5%) consented to participate. Reasons for not taking part were no interest (three midwives) and worries about potential conflicts with her employer (one midwife). As some midwives gave more than one class, 50 antenatal classes were included. On average, a class comprised 6.9 participants (+/- SD 2.5; minimum 2, maximum 12 participants). After being informed about the study, 344 (93.0%) of 370 eligible women consented to participate. Following written informed consent, the questionnaires were distributed during antenatal classes. Data collection occurred over a one year period from 2000 to 2001. The study was approved by the ethical committee of the University of Heidelberg in May 2000.

### Questionnaires

The Cambridge Worry Scale (CWS) is a self-administered questionnaire for assessing the content and extent of worries in pregnancy [12]. It contains items concerning such issues as the baby's health and giving birth. Each item is scored on a six-point Likert-type scale ranging from *not a worry *(0) to *major worry *(5). The CWS scale can be used throughout pregnancy. Depending on the pregnancy week, additional context-specific items can be added or removed as appropriate. Similar to the CWS used in mid-pregnancy in the "Cambridge Prenatal Screening study", the questionnaire used in this study comprised 17 items (see additional file 1), which allows the calculation of a total sum score that ranges from 0 to 85. An open-ended question at the end of the questionnaire gives respondents the opportunity to report other concerns not included in the scale. Two native German speakers carried out independent translations of the CWS from English to German. The forward translations were compared with each other and with the original English version. After discussing any discrepancies, the two versions were synthesized to form one common German version. The scales were then back-translated independently by two native English speakers whose second language was German.

The questionnaire used in this study also included the German version of the Spielberger State-Trait-Inventory (STAI). The STAI consists of two, 20-item questionnaires, each measuring a different dimension of anxiety (state anxiety and trait anxiety) [9]. The first set of statements (state anxiety) measures how the respondent currently feels. It represents a transitory emotional state that can fluctuate over time and vary in intensity, depending on the situation. The second set of statements (trait-anxiety) assesses how the respondent feels in general, i.e. the individual level of anxiety proneness. This characteristic is considered to be stable over time. Each item is scored on a 4-point intensity scale, with a total score that ranges from 20 to 80 for state-, and the same for trait-anxiety (high scores indicate more severe anxiety) [9].

Additionally, the questionnaire contained questions on obstetric history, previous antenatal consultations and related test results, and smoking habits. The questionnaire also included a modified version of the Soziodat Inventory, a 10-item self-report questionnaire for socio-demographic data developed by Brähler et al. (Brähler E, Felder H, Florin I, Tuschen B. *Soziodemographischer Fragebogen Soziodat*, 1993. Leipzig: Unpublished paper). Clinical data were obtained by reviewing and analyzing participants' antenatal records (so-called *Mutterpass*, literally passport for mothers). In Germany, every pregnant woman receives a *Mutterpass *that contains all screening test results [28].

### Pretest of the Cambridge Worry Scale

A group of 21 pregnant women recruited from three antenatal classes were given the prefinal version of the CWS to complete. They were briefly interviewed in order to check that they understood each question and the choice of responses. They were also asked for their general comments on the questionnaire. All the findings were evaluated to assess face validity.

The general comments of the 21 women who pretested the questionnaire indicated that the wording was easy to understand and the layout was good. The pretest confirmed that many women did not know how to score the response to the item "giving up work". In fact, many women had already given up work before pregnancy or had never worked at all. In consequence, we kept the term "if applicable" in parenthesis, comparable to the original CWS. The results of the pretest led to revisions and some cultural adaptations of the items but were not included in the statistical analyses.

### Statistical analyses

We calculated mean values of the single CWS items by averaging the answers on the Likert scale, with the corresponding 95% confidence intervals (95% CI) and standard deviations. We also calculated the mean values and standard deviations of the total sum scores of the CWS and STAI. We used the Mann-Whitney test for bivariate analyses, and the Cronbach's α coefficient of reliability to assess the internal consistency of the CWS, employing data from all items of the CWS. An α value < 0.80 can be considered as low, 0.80 - 0.90 as satisfactory and > 0.90 as high [29]. Exploratory factor analysis (EFA) was performed on the CWS scale with the four items "problems with the law", "giving up work", "whether your partner will be at the birth" and "the possibility of going into labour too early" removed in order to be consistent with previous factor analyses of the CWS [12,15] and as the item "giving up work" did not apply equally to all participants.

Principle component analysis was performed using oblique rotation (with Eigenvalues > 1) [30], as performed by other authors when validating the original English version of the scale [12,15]. Tests on sampling adequacy (Kaiser-Meyer-Olkin-criterion) and multicollinearity (Bartlett test of sphericity) were undertaken prior to factor extraction to ensure that the scale items were appropriate for principle component analysis. A Kaiser-Meyer-Olkin-criterion ≥ 0.50 and a Bartlett test of sphericity with p < 0.05 were regarded as mandatory for factor analysis [30]. For convergent validity, the Spearman rank correlation coefficient was used to assess the relationship between the total sum score/factor scores of the CWS and the sum scores of the state-, and of the trait-anxiety questionnaires of Spielberger. All p values were 2-sided and reported as being statistically significant on the basis of a significance level of 0.05. Statistical analyses were performed using SPSS, version 15 [31].

## Results

### Description of the study population

Table 1 displays the socio-demographic characteristics of the study population. In order to assess the representativeness of the sample, background characteristics of the study population were compared to the child-bearing population in the federal state of Baden-Württemberg [32]. An examination of the characteristics of the two groups showed no differences in age, smoking habits, obstetric risk factors and outcomes. However, women of non-German nationality and housewives were underrepresented, and nulliparae and skilled workers overrepresented in the study sample. According to the statistics office in Baden-Württemberg, the total number of live-births in the regions of interest was 8636 in the year 2001 [33]. Compared to the annual statistic, the births included in this study (n = 344) comprised at least 4% of all births in the investigated area in the same year and represented around 12% of pregnancies during the four month recruitment period [33]. The mean gestational week was 31.4 (SD 2.7), the overall mean score for trait anxiety was 36.4 (SD 8.7).

**Table 1:** Characteristics of the study sample compared to the pregnant population of Baden-Württemberg

Sociodemographic and obstetric characteristics	Study population (n = 344)		Pregnant population of Baden-Württemberg in 2001 (n = 86 849)
	**n**	**%**	**%**

**Age (years)**			
< 18	1	0.3	0.5
18-34	280	81.4	78.8
> 34	63	18.3	20.8
			
**Nationality**			
German	314	91.3	79.0
Other	30	8.7	21.0
			
**Occupation^a^**			
Housewife	70	20.9	45.4^b^
Trainee/student	15	4.5	2.5^b^
Unskilled worker	11	3.3	4.4^b^
Skilled worker/civil servant	207	61.8	35.0^b^
Executive position	32	9.6	12.7^b^
			
**Gravidity**			
First	200	58.1	38.8
> 1	144	41.9	61.2
			
**Parity**			
0	229	66.6	46.4
≥1	115	33.4	53.6
			
**Risk factors documented in clinical data^a^**			
Yes	151	61.6	63.4
No	94	38.4	36.6
			
**Smoking during pregnancy^a^**			
Yes	36	11.2	9.5
No	285	88.8	90.5
			
**Mode of delivery^a^**			
Vaginal	224	68.9	70.0
Cesarean section	76	23.4	23.5
Forceps or vacuum	25	7.7	6.4

^a ^Members sum < 344 due to missing data

^b ^These data refer to the perinatal statistics of Baden-Württemberg in 2004 (n = 76 803) because data for 2001 were incomplete

### Content and extent of reported worries

Table 2 displays the mean values with corresponding 95% confidence intervals for each of the items of the CWS. The major worries referred to the process of giving birth (CWS mean value 2.26) and the possibility that something might be wrong with the baby (1.99), followed by worries about coping with the new baby (1.57), going to hospital (1.29) and the possibility of going into labour too early (1.28). The item with the lowest mean value concerned problems with the law (0.15).

**Table 2:** Descriptive parameters of the German Cambridge Worry Scale

	n	0 Not a worry	1	2	3	4	5 Major worry	Mean value [95% CI]
**Item**		**%**	**%**	**%**	**%**	**%**	**%**	

Your housing	344	67.7	11.6	6.7	8.4	4.1	1.5	0.74 [0.60; 0.87]
Money problems	344	48.0	17.7	15.4	11.3	4.1	3.5	1.16 [1.01;1.31]
Problems with the law	344	93.3	2.9	0.9	1.7	0.9	0.3	0.15 [0.08; 0.22]
Your relationship with your husband/partner	342	77.8	11.7	3.5	3.5	2.0	1.5	0.45 [0.34;0.56]
Your relationship with your family and friends	342	76.9	11.7	6.1	3.8	0.3	1.2	0.42 [0.32; 0.52]
Your own health	343	60.6	21.0	12.2	3.5	2.0	0.6	0.67 [0.56; 0.78]
The health of someone close to you	342	66.1	7.3	11.1	8.8	3.2	3.5	0.86 [0.71; 1.01]
Employment problems	339	73.7	9.7	6.2	4.7	3.2	2.4	0.61 [0.48; 0.74]
The possibility of something being wrong with the baby	342	16.7	25.1	21.6	21.1	10.2	5.3	1.99 [1.84; 2.14]
Going to hospital	342	43.3	17.8	19.3	9.9	5.6	4.1	1.29 [1.14; 1.44]
Internal examinations	343	72.9	15.7	7.0	3.2	0.6	0.6	0.45 [0.35; 0.54]
Giving birth	342	14.3	17.3	26.6	21.1	11.4	9.4	2.26 [2.10; 2.42]
Coping with the new baby	343	27.4	23.0	25.9	14.9	6.4	2.3	1.57 [1.43; 1;71]
Giving up work	277	51.3	18.4	12.6	10.1	4.7	2.9	1.07 [0.91; 1.24]
Whether your partner will be with you for the birth	337	75.7	9.2	4.7	5.9	2.4	2.1	0.56 [0.44; 0.69]
The possibility of miscarriage	332	56.3	18.1	11.4	6.3	2.7	5.1	0.96 [0.81; 1.12]
The possibility of going into labour too early	329	42.2	20.4	19.1	9.1	3.6	5.5	1.28 [1.12; 1.44]

Each item was given the full range of scores from 0 to 5, with zero the modal response for 15 of the 17 items. Due to the optional character, the item "giving up work" presented a high percentage of missing values, with only 277 (80.5%) women answering it. A total of 22 (6.4%) participants added other concerns (besides the 17 items) in response to the open-ended question, indicating that there were further concerns that mattered to them. Some women reported worries under this category that were already listed in the CWS, but in most cases respondents further specified their worries (e.g. worry that something might happen to the baby during birth). Some women made comments about aspects not included in the questionnaire, such as the reaction of older children to the new baby (n = 5), the possibility of a cesarean section (n = 3), legal aspects related to the name/status of the newborn (n = 2) and logistic problems regarding the care of older children (n = 2).

The scale of 17 items exhibited satisfactory internal consistency with Cronbach's α coefficient of reliability measuring 0.80.

There was no statistically significant difference between the total sum scores of the CWS by age-group or gravidity. However, nulliparous women were slightly more worried than women with childbearing experience (mean CWS sum score of 16.5 versus 13.9; p < 0.05, Mann-Whitney-test).

### Factor analysis

The tests of sampling adequacy showed a meritorious correlation of items (Kaiser-Meyer-Olkin-criterion = 0.75). The Bartlett test of sphericity was highly significant (p < 0.001), which confirmed the prerequisite for factor analysis. A principle component analysis with oblique rotation revealed that four factors accounted for 55.4% of the total variance (factor structure shown in Table 3). We classified the four factors as socio-medical (26.6%), socio-economic and relationships (12.8%), health of the baby (8.1%), health of mother/other (7.9%).

**Table 3:** Factor structure of the German CWS

Factors of the German CWS	Factor loading
**Socio-medical (26.6%^a^)**	
Going to hospital	0.83
Internal examinations	0.65
Giving birth	0.73
Coping with the new baby	0.58
	
**Socio-economic and relationships (12.8%^a^)**	
Your housing	0.69
Money problems	0.74
Your relationship with your husband/partner	0.61
Your relationship with your family and friends	0.44
Employment problems	0.57
	
**Health of the baby (8.1%^a^)**	
The possibility of something being wrong with the baby	0.66
The possibility of miscarriage	0.76
	
**Health of mother/other (7.9%^a^)**	
Your own health	0.51
The health of someone close to you	0.87

^a ^Percentage of the total variance explained by the factor

### Convergent validity

**Figure 1:**
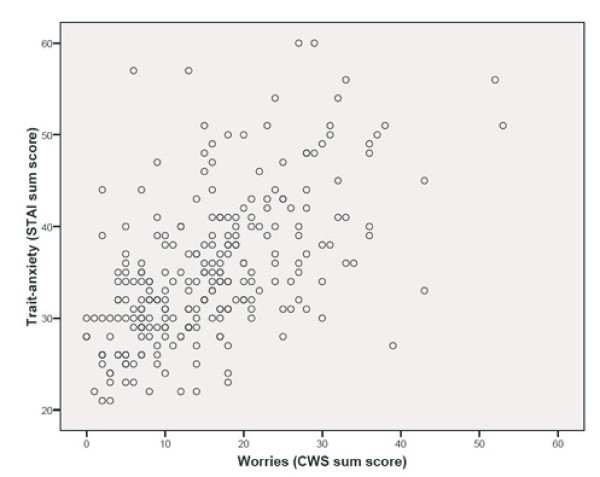
**Correlation between trait-anxiety and worries^a^**. ^a ^Spearman rank correlation coefficient.

**Figure 2:**
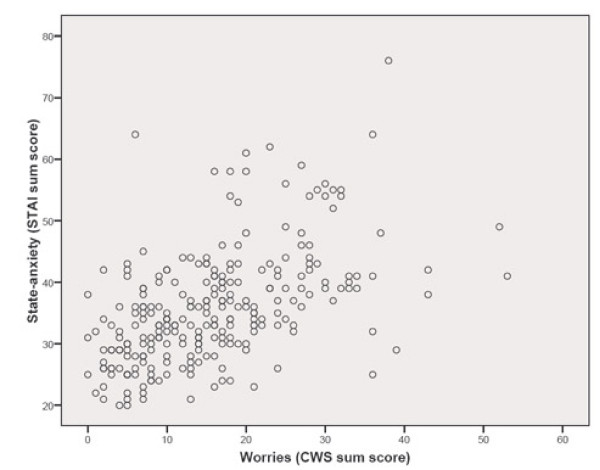
**Correlation between state-anxiety and worries^a^**. ^a ^Spearman rank correlation coefficient.

We found a statistically significant moderate correlation between the sum score of the CWS and trait anxiety (r = 0.60; figure 1), as well as between the sum score of the CWS and state anxiety (r = 0.56; figure 2). Table 4 displays the correlations of the factor scores of the CWS and the state-, and trait-anxiety. All CWS factor scores correlated statistically significantly with both state and trait anxiety. The highest correlation was between the socio-medical factor and the state- (r = 0.52), and trait-anxiety scores of the STAI (r = 0.53). The lowest correlation was found between the baby's health factor and trait anxiety (r = 0.18).

**Table 4:** Correlations between factors of the CWS and state-/trait-anxiety^a^

	Socio-medical	Socio-economic and relationships	Health of the baby	Health of mother/other	State-anxiety	Trait-anxiety
Socio-medical	1.0					
Socio-economic and relationships	0.26*	1.0				
Health of the baby	0.26*	0.19*	1.0			
Health of mother/other	0.26*	0.15*	- 0.05	1.0		
State-anxiety	0.52*	0.29*	0.22*	0.27*	1.0	
Trait-anxiety	0.53*	0.40*	0.18*	0.31*	0.70*	1.0

^a ^Spearman rank correlation coefficient; n (max) = 325 participants

* Statistically significant (p < 0.05)

## Discussion

This study showed that the major worries reported by pregnant women were about giving birth and that something might be wrong with the baby. The internal consistency of the scale (0.80) was satisfactory, and we found a four-factor structure, similar to previous studies.

The study population was representative for the child-bearing population in the federal state of Baden-Württemberg with regard to age, smoking habits, obstetric risk factors and outcomes. Despite the fact that costs for antenatal classes are covered by medical insurance and most pregnant women take part in these classes [27], women of non-German nationality and housewives were underrepresented and skilled workers overrepresented, reflecting the relatively urban study region. In addition, nulliparae were overrepresented, which can be explained by the fact that multiparae already have child-bearing experience and thus participation rate in these classes is lower.

The overall mean score for trait anxiety (36.4) was comparable to the mean trait-anxiety scores in other studies of pregnant women, indicating that our sample was not generally more anxious than other similar populations: In a study by Green et al. the mean trait anxiety was 38.4 (+/- SD 8.1) in the 16^th ^gestational week [14] and in a study by Georgsson Öhman et al. it was 34.0 (+/- SD 8.5) in the 24^th ^gestational week [18].

This study showed that worries relating to the birth and to the possibility that something might be wrong with the baby were the major worries for participants. The worry relating to the baby's health ranked second in our study, whereas in comparable studies it ranked top [12,14,16]. The worry about the baby's health is strongly influenced by antenatal care, and women have high expectations of antenatal care in terms of possibilities for preventing fetal morbidity [34]. Most women take part in screening programs to be reassured that the baby is healthy and pregnancy is progressing normally [34,35]. This is true for ultrasound examinations [36], and for other tests such as serum screening [37]. However, contrary to their expectations of reassurance, many women report suspicious findings in antenatal care, which may lead to further examinations and cause or increase worries [8].

An additional concern for women - not included as an item in the CWS used in this study - was worry about the reaction of older children to the newborn. This concern - already identified by Green et al. - can be added as an item if the scale is to be used in late pregnancy [12]. Women did not mention additional concerns regarding maternity services, contrary to the Swedish women that participated in the study by Georgsson Öhman et al. [16]. The findings of Georgsson Öhman et al. were probably specific for the Stockholm region at the time of the study, where two of six maternity units had closed for financial reasons [16].

The pretest of the questionnaire indicated that many women did not know how to score the response to the item "giving up work", probably because many women had already given up work before pregnancy or had never worked at all. However, participants may also have found it difficult to complete this item because it might reflect gender stereotypes and intrinsically devaluate the unpaid work of mothering. Further research should focus on whether it might be more appropriate to ask specifically about income security or maternity leave, as these factors also influence maternal psychological well-being. Cooklin et al., for instance, showed that nearly 18% of women experience pregnancy-related workplace discrimination or difficulty in negotiating maternity leave, and that experiencing adversity in the workplace during pregnancy was associated with increased depression and anxiety [38].

Cronbach's α coefficient for the German version of the CWS (0.80) was satisfactory and comparable to those reported for the original scale by Green et al. (between 0.76 and 0.79) [12] and that reported by Jomeen and Martin (0.80) [15]. Georgsson Öhman et al. also registered a similar α-value (0.81) for the Swedish CWS [16].

The principle component analysis revealed a four-factor structure, similar to the four-factor structure identified by Green et al. [12] and to the five-factor structure found by Jomeen and Martin [15]. The primary factor identified in this study on the socio-medical aspects of having a baby was consistent with that of the two studies reported above [12,15]. We found that items concerning the baby's health and maternal and others' health loaded on two single factors, consistent with the findings of Jomeen and Martin [15], whereas in the study of Green these items loaded on one common factor [12]. Jomeen and Martin noted that the loading of health factors on two distinct factors appears to be commensurate with two health concepts, those of the health of the baby and those of others' health or "non-baby health" [15], which is confirmed by this study.

Items concerning socio-economic and relationship aspects loaded on a single factor in this study, whereas in previous studies they loaded on two separate factors [12,15]. This difference is probably attributable to country-specific aspects. One way to interpret the difference is to take into account that in Germany the socio-economic situation of married couples may differ from Great Britain. One reason, for instance, can be found in the taxation system. While most countries rely on individual taxation, in Germany married couples can apply for joint taxation. This taxation system has been criticized as being a fiscal disincentive to the full-time employment of second-earners [39]. In fact, Germany represents one of the countries with the lowest share of households with two partners in full-time employment in Europe. The traditional male breadwinner model is still relatively common (in more than 40% of households), particularly in families with children [39]. However, such considerations require further research.

Convergent validity was examined by investigating the association between the total sum scores and the factor scores of the CWS and the state and trait anxiety of the STAI. We found a statistically significant moderate correlation between the total worry score and trait anxiety (r = 0.60). This represents moderate agreement, which shows that the CWS assesses a slightly different construct to the trait-anxiety questionnaire. This is important since it confirms that the CWS scores are not simply attributable to anxiety proneness [12]. Green et al. found a similar correlation between total CWS and trait anxiety [12]. Jomeen and Martin found a correlation of r = 0.38 between total CWS scores and anxiety [15], although the comparison is hampered by the fact that Jomeen and Martin used a different instrument, the Hospital Anxiety and Depression Scale for measuring anxiety [15]. We also found that the single factors of the CWS represented a different construct compared with state and trait anxiety but had the necessary overlap to be externally valid. The highest correlation was between the socio-medical factor and the state-, and trait-anxiety scores of the STAI. This might reflect the fact that the socio-medical factor integrates several aspects of anxiety that are covered by the STAI. Green et al. found a similar range of correlations between the factors assessed in the CWS and those in the STAI [12]. However, because of the different factor structure the findings of Green et al. are not directly comparable to the findings of this study.

This study has some limitations, attributable to the use of a cross-sectional design with a one-point measurement. The psychometric properties of the CWS described in this publication refer to a sample of women with a mean pregnancy week of 31. In the course of pregnancy, the extent of worries can be described as U-shaped, with a decrease in mid-pregnancy and an increase as birth approaches [14,16,40]. Thus, assessing the psychometric properties of the CWS on women in earlier or later pregnancy would probably lead to somewhat different findings.

The CWS is a flexible, context-specific tool which has allowed its adaptation for use in studies with other populations, such as parents of disabled children [25] and women with a family history of cancer [26]. In all of these, some of the core items such as money and housing were retained and pilot studies enabled the other main areas of concern to be adapted to suit the target group [12]. From a public health perspective, the CWS has considerable potential to be used as a context-specific, user-friendly tool in various populations. Further research is required to assess whether its use might also be useful to clinicians to better address women's concerns.

## Conclusions

This study showed that the major concerns of pregnant women were related to worries about birth and the possibility that something might be wrong with the baby. The German version of CWS was well understood and easy to fill in. Internal consistency was satisfactory, and we found a four-factor structure, similar to previous studies. Tests of convergent validity showed that the German CWS represents a different construct when compared with state and trait anxiety but has the desired overlap. The German version of CWS represents a valuable tool for use in scientific studies and is also likely to be useful to clinicians.

## Competing interests

The authors declare that they have no competing interests.

## Authors' contributions

JJP and AJ designed the study and collected the data. JJP, MAP, CG performed the statistical analyses. JJP and MAP drafted the manuscript, and all authors participated in the interpretation of the findings, reviewed the manuscript, and approved the final manuscript.

## Pre-publication history

The pre-publication history for this paper can be accessed here:

http://www.biomedcentral.com/1471-2458/9/490/prepub

## Supplementary Material

Additional file 1 **German version of the Cambridge Worry Scale**. Additional file 1 contains the German version of the Cambridge Worry Scale used in this study. Click here for file

## References

[B1] Lukesch H Lukesch H Verbreitung von Schwangerschafts- und Geburtsängsten [Distribution of pregnancy- and birth-related anxiety] Schwangerschafts- und Geburtsängste. Verbreitung - Genese - Therapie [Pregnancy- and birth-related anxiety. Distribution - genesis - therapy] 1981 Stuttgart: Ferdinand Enke Verlag 18 29

[B2] Teixeira JMA Fisk NM Glover V Association between maternal anxiety in pregnancy and increased uterine artery resistance index: cohort based study BMJ 1999 318 153 157 9888905 10.1136/bmj.318.7177.153 PMC27690

[B3] Alder J Fink N Bitzer J Hosli I Holzgreve W Depression and anxiety during pregnancy: a risk factor for obstetric, fetal and neonatal outcome? A critical review of the literature J Matern Fetal Neonatal Med 2007 20 189 209 10.1080/14767050701209560 17437220

[B4] Van den Bergh BR Mulder EJ Mennes M Glover V Antenatal maternal anxiety and stress and the neurobehavioural development of the fetus and child: links and possible mechanisms. A review Neurosci Biobehav Rev 2005 29 237 258 10.1016/j.neubiorev.2004.10.010 15811496

[B5] Kowalcek I Lammers C Brunk J Bieniakiewicz I Gembruch U Angst der Schwangeren vor und nach der pränatalen Untersuchung bei unauffälligen und bei auffälligen Befunden [Fears of pregnant women if prenatal examination yields or does not yield any findings] Zentralbl Gynakol 2002 124 170 175 10.1055/s-2002-32263 12070797

[B6] Green JM Hewison J Bekker HL Bryant LD Cuckle HS Psychosocial aspects of genetic screening of pregnant women and newborns: a systematic review Health Technol Assess 2004 8 1 109 10.3310/hta8330 15298822

[B7] Götzmann L Schönholzer SM Kölble N Klaghofer R Scheuer E Zimmermann R Die Verdachtsdiagnose einer fetalen Entwicklungsstörung in der Ultraschall-Untersuchung: Auswirkungen auf das psychische Befinden schwangerer Frauen [Suspected fetal malformation in ultrasound examination: effects on the psychological well-being of pregnant women] Ultraschall Med 2002 23 33 40 10.1055/s-2002-20073 11842370

[B8] Petersen J Jahn A Suspicious findings in antenatal care and their implications from the mothers' perspective: a prospective study in Germany Birth 2008 35 41 49 10.1111/j.1523-536X.2007.00210.x 18307487

[B9] Laux L Glanzmann P Schaffner P Spielberger CD Das State-Trait-Angstinventar. Theoretische Grundlagen und Handanweisung [The State-Trait-Anxiety Inventory. Theoretical Basics and Instructions] 1981 Weinheim: Beltz Test

[B10] Grant KA McMahon C Austin MP Maternal anxiety during the transition to parenthood: a prospective study J Affect Disord 2008 108 101 111 10.1016/j.jad.2007.10.002 18001841

[B11] Fertl KI Bergner A Beyer R Klapp BF Rauchfuss M Levels and effects of different forms of anxiety during pregnancy after a prior miscarriage Eur J Obstet Gynecol Reprod Biol 2009 142 23 29 10.1016/j.ejogrb.2008.09.009 18986753

[B12] Green JM Kafetsios K Statham H Snowdon C Factor structure, validity and reliability of the Cambridge Worry Scale in a pregnant population J Health Psychol 2003 8 753 764 10.1177/13591053030086008 14670208

[B13] Hundley V Gurney E Graham W Rennie AM Can anxiety in pregnant women be measured using the State-trait-Anxiety Inventory Midwifery 1998 14 118 121 10.1016/S0266-6138(98)90009-2 10382481

[B14] Statham H Green JM Kafetsios K Who worries that something might be wrong with the baby? A prospective study of 1072 pregnant women Birth 1997 24 223 233 10.1111/j.1523-536X.1997.tb00595.x 9460313

[B15] Jomeen J Martin CR The factor structure of the Cambridge Worry Scale in early pregnancy Journal of prenatal & perinatal psychology & health 2007 20 25 48

[B16] Georgsson Öhman S Grunewald C Waldenstrom U Women's worries during pregnancy: testing the Cambridge Worry Scale on 200 Swedish women Scand J Caring Sci 2003 17 148 152 10.1046/j.1471-6712.2003.00095.x 12753515

[B17] Sikorski J Wilson J Clement S Das S Smeeton S A randomised controlled trial comparing two schedules of antenatal visits: the antenatal care project BMJ 1996 312 546 553 8595286 10.1136/bmj.312.7030.546 PMC2350357

[B18] Georgsson Öhman S Saltvedt S Grunewald C Waldenstrom U Does fetal screening affect women's worries about the health of their baby? A randomized controlled trial of ultrasound screening for Down's syndrome versus routine ultrasound screening Acta Obstet Gynecol Scand 2004 83 634 640 10.1111/j.0001-6349.2004.00462.x 15225187

[B19] Waldenstrom U Hildingsson I Rubertsson C Radestad I A negative birth experience: Prevalence and risk factors in a national sample Birth 2004 31 17 27 10.1111/j.0730-7659.2004.0270.x 15015989

[B20] Hildingsson I Radestad I Swedish women's satisfaction with medical and emotional aspects of antenatal care J Adv Nurs 2005 52 239 249 10.1111/j.1365-2648.2005.03584.x 16194177

[B21] Georgsson Öhman S Grunewald C Waldenstrom U Perception of risk in relation to ultrasound screening for Down's syndrome during pregnancy Midwifery 2007 25 264 276 10.1016/j.midw.2007.04.007 17920172

[B22] Schytt E Waldenstrom U Risk factors for poor self-rated health in women at 2 months and 1 year after childbirth J Womens Health (Larchmt) 2007 16 390 405 10.1089/jwh.2006.0030 17439384

[B23] Jomeen J Martin CR The impact of choice of maternity care on psychological health outcomes for women during pregnancy and the postnatal period J Eval Clin Pract 2008 14 391 398 10.1111/j.1365-2753.2007.00878.x 18373580

[B24] Georgsson OS Grunewald C Waldenstrom U Perception of risk in relation to ultrasound screening for Down's syndrome during pregnancy Midwifery 2009 25 264 276 10.1016/j.midw.2007.04.007 17920172

[B25] Green JM Murton FE Duchenne muscular dystrophy: Families' responses to diagnosis and genetic counselling 1993 Centre for Family Research University of Cambridge

[B26] Collins V Halliday J Warren R Williamson R Cancer worries, risk perceptions and associations with interest in DNA testing and clinic satisfaction in a familial colorectal cancer clinic Clin Genet 2000 58 460 468 10.1034/j.1399-0004.2000.580606.x 11149615

[B27] Siegmund-Schultze E Kielblock B Bansen T Schwangerschaft und Geburt: Was kann die Krankenkasse tun? Eine sozioökonomische Analyse der Bedürfnisse von KKH-versicherten Frauen in Bezug auf Schwangerschaft, Geburt und Babyzeit [Pregnancy and childbirth: what can a health plan do? A socio-economic analysis of the needs among KKH-members in matters of pregnancy, childbirth, and the first year of childhood] Gesundh ökon Qual manag 2008 13 210 215 10.1055/s-2008-1027223

[B28] Gemeinsamer Bundesausschuss (GBA) Richtlinien des Bundesausschusses der Ärzte und Krankenkassen über die ärztliche Betreuung während der Schwangerschaft und nach der Entbindung - "Mutterschaftsrichtlinien" [Guidelines for obstetricians for the provision of ante- and postnatal care - "German maternity care guidelines"] http://www.g-ba.de/downloads/62-492-389/RL_Mutter-2009-08-06.pdf

[B29] Bühner M Einführung in die Test- und Fragebogenkonstruktion 2006 2 München: Pearson Studium

[B30] Backhaus K Erichson B Plinke W Weiber R Multivariate Analysemethoden: Eine anwendungsorientierte Einführung [Multivariate analysis methods: an application-oriented introduction] 2006 11 Berlin: Springer

[B31] Statistical Package for the Social Sciences SPSS for Windows [Computer program] Version 15. Chicago: Author; 2007

[B32] Geschäftsstelle Qualitätssicherung im Krankenhaus bei der Baden-Württembergischen Krankenhausgesellschaft Qualitätssicherung Geburtshilfe - Jahresauswertung Baden-Württemberg [Quality assurance obstetrics - yearly evaluation 2001, Baden-Württemberg] http://www.geqik.de/fileadmin/Archiv/2001/perinatologie/modul_16-1/gesamtstatistik.pdf

[B33] Statistisches Landesamt Baden-Württemberg Struktur- und Regionaldatenbank [Structural and regional database] http://www.statistik.baden-Wuerttemberg.de/SRDB/home.asp?H=1&U = 04&T=01065011&E=KR

[B34] Hildingsson I Waldenstrom U Radestad I Women's expectations on antenatal care as assessed in early pregnancy: number of visits, continuity of caregiver and general content Acta Obstet Gynecol Scand 2002 2 118 25 11942901

[B35] Green JM Abramansky L, Chapple I Women's experiences of prenatal screening and diagnosis Prenatal diagnosis-The human side 1994 37 53

[B36] Eurenius K Axelsson O Gallstedt-Fransson I Sjoden PO Perception of information, expectations and experiences among women and their partners attending a second-trimester routine ultrasound scan Ultrasound Obstet Gynecol 1997 9 86 90 10.1046/j.1469-0705.1997.09020086.x 9132261

[B37] Santalahti P Aro AR Hemminki E Helenius H Ryynanen M On what grounds do women participate in prenatal screening? Prenat Diagn 1998 18 153 165 10.1002/(SICI)1097-0223(199802)18:2<153::AID-PD240>3.0.CO;2-Z 9516017

[B38] Cooklin AR Rowe HJ Fisher JR Employee entitlements during pregnancy and maternal psychological well-being Aust N Z J Obstet Gynaecol 2007 47 483 490 10.1111/j.1479-828X.2007.00784.x 17991114

[B39] Dingeldey I European tax systems and their impact on family employment patterns Jnl Soc Pol 2001 30 653 672

[B40] Gloger-Tippelt G Gloger-Tippelt G Schritte des Übergangs zur Elternschaft I: Schwangerschaft Schwangerschaft und erste Geburt: Psychologische Veränderungen der Eltern [Pregnancy and first birth: Psychological changes of the parents] 1988 Stuttgart, Berlin, Köln, Mainz: Kohlhammer 59 91

