# Supplementary material for: A survey on worries of pregnant women - testing the German version of the Cambridge Worry Scale
Source: BMC Public Health. 2009 Dec 28;9:490. doi: 10.1186/1471-2458-9-490 (PMC2811709; doi:10.1186/1471-2458-9-490)
Supplement: Additional file 1 — German version of the Cambridge Worry Scale. Additional file 1 contains the German version of the Cambridge Worry Scale used in this study. [file 1471-2458-9-490-S1.DOC]

**Additional file 1 – German version of the Cambridge Worry Scale**

| Die meisten Menschen machen sich wegen irgendetwas Sorgen. Ziel dieses Fragebogens ist es nicht, Ihnen weitere Dinge aufzuzeigen, wegen der Sie sich Sorgen machen könnten. Wir würden einfach nur gerne von Ihnen wissen, ob die unten genannten Aspekte Ihnen überhaupt Sorgen bereiten. Bitte kreuzen Sie eine Zahl zwischen 0 und 5 an, um anzugeben, inwieweit Ihnen jeder dieser Punkte zurzeit Sorgen bereitet: 0 bedeutet dabei, dass Ihnen der Punkt keine Sorgen und 5 dagegen, dass er Ihnen sehr große Sorgen bereitet. | | | | | | |
| --- | --- | --- | --- | --- | --- | --- |
|  | | | | | | |
| **keine Sorgen große Sorgen** | | | | | | |
| 1. Ihre Wohnverhältnisse | 0 | 1 | 2 | 3 | 4 | 5 |
|  | | | | | | |
| 2. Finanzielle Situation | 0 | 1 | 2 | 3 | 4 | 5 |
|  | | | | | | |
| 3. Konflikte mit dem Gesetz | 0 | 1 | 2 | 3 | 4 | 5 |
|  | | | | | | |
| 4. Ihre Beziehung zu Ihrem Ehemann/ Partner | 0 | 1 | 2 | 3 | 4 | 5 |
|  | | | | | | |
| 5. Ihre Beziehung zu Ihrer Familie und Freunden | 0 | 1 | 2 | 3 | 4 | 5 |
|  | | | | | | |
| 6. Ihre eigene Gesundheit | 0 | 1 | 2 | 3 | 4 | 5 |
|  | | | | | | |
| 7. Die Gesundheit von jemandem, der Ihnen nahe  steht | 0 | 1 | 2 | 3 | 4 | 5 |
|  | | | | | | |
| 8. Probleme mit der Arbeit | 0 | 1 | 2 | 3 | 4 | 5 |
|  | | | | | | |
| 9. Die Möglichkeit, dass etwas mit Ihrem Kind  nicht in Ordnung sein könnte | 0 | 1 | 2 | 3 | 4 | 5 |
|  | | | | | | |
| 10. Die Entbindungsklinik/ das Geburtshaus | 0 | 1 | 2 | 3 | 4 | 5 |
|  | | | | | | |
| 11. Ärztliche Untersuchungen | 0 | 1 | 2 | 3 | 4 | 5 |
|  | | | | | | |
| 12. Die Geburt selbst | 0 | 1 | 2 | 3 | 4 | 5 |
|  | | | | | | |
| 13. Der Umgang mit dem neuen Baby | 0 | 1 | 2 | 3 | 4 | 5 |
|  | | | | | | |
| 14. Vorübergehende oder dauerhafte Aufgabe der  Berufstätigkeit *(falls hierzu keine Angaben*  *möglich sind, Frage bitte überspringen)* | 0 | 1 | 2 | 3 | 4 | 5 |
|  | | | | | | |
| 15. Ob Ihr Partner bei der Geburt anwesend sein  wird | 0 | 1 | 2 | 3 | 4 | 5 |
|  |  |  |  |  |  |  |
| 16. Die Möglichkeit einer Fehlgeburt | 0 | 1 | 2 | 3 | 4 | 5 |
|  |  |  |  |  |  |  |
| 17. Die Möglichkeit vorzeitiger Wehen | 0 | 1 | 2 | 3 | 4 | 5 |
|  | | | | | | |
| Wenn es noch etwas anderes gibt, dass Ihnen Sorgen bereitet, oder wenn Sie etwas ergänzen möchten, können Sie es gerne hier eintragen: | | | | | | |
